# Advancing atmospheric solids analysis probe mass spectrometry applications: a multifaceted approach to optimising clinical data set generation†

**DOI:** 10.1039/d5an00166h

**Published:** 2025-05-12

**Authors:** Liwen Song, Jasmine G. Reese, Michael A. Platt, Claire Lewis, Annabel S. J. Eardley-Brunt, Bo Sun, Olaf Ansorge, Claire Vallance

**Affiliations:** a Department of Chemistry, University of Oxford, Chemistry Research Laboratory 12 Mansfield Rd Oxford OX1 3TA UK claire.vallance@chem.ox.ac.uk; b Academic Unit of Neuropathology, Nuffield Department of Clinical Neurosciences, University of Oxford, John Radcliffe Hospital Oxford OX3 9DU UK

## Abstract

The use of rapid mass spectrometry techniques, such as atmospheric-solids-analysis-probe mass spectrometry (ASAP-MS), in the analysis of metabolite patterns in clinical samples holds significant promise for developing new diagnostic tools and enabling rapid disease screening. The rapid measurement times, ease of use, and relatively low cost of ASAP-MS makes it an appealing option for use in clinical settings. However, despite the potential of such approaches, a number of important experimental considerations are often overlooked. As well as instrument-specific choices and settings, these include the treatment of background noise and/or contaminant peaks in the mass spectra, and the influence of consumables, different users, and batch effects more generally. The present study assesses the impact of these various factors on measurement accuracy and reproducibility, using human brain and cerebrospinal fluid samples as examples. Based on our results, we make a series of recommendations relating to optimisation of measurement and cleaning protocols, consumable selection, and batch effect detection and correction, in order to optimise the reliability and reproducibility of ASAP-MS measurements in clinical settings.

## Abstract 2

We assess the impact of various experimental factors on ASAP-MS spectra, and make a series of recommendations to optimise the reliability and reproducibility of such measurements in clinical settings.

## Graphical abstract

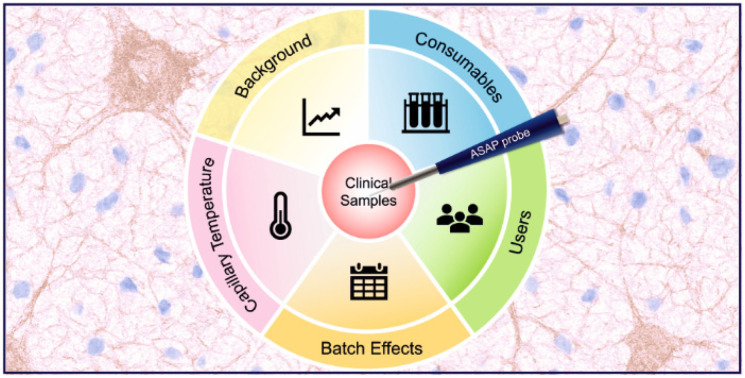

## 1 Introduction

Metabolomics studies aim to achieve quantitative analysis of some or all metabolites within an organism, and to establish relationships between the metabolite profile and physiological or pathological changes.^1^ The analysis focuses on small molecules with a molecular weight of less than 1000 Da. Depending on the research objectives, a metabolomics study may use a *targeted* or *untargeted* approach. Untargeted metabolomics aims to detect and analyse all small-molecule metabolites, while targeted approaches focus on those associated with specific metabolic pathways of interest.^2^ Within clinical research, mass-spectrometry-based metabolomics coupled with machine learning techniques has emerged as a powerful approach for biomarker discovery, disease diagnosis, and understanding the underlying mechanisms of various pathological conditions.^3^ The atmospheric solids analysis probe (ASAP) is employed within an atmospheric pressure chemical ionisation (APCI) source, and offers a route to rapid analysis of volatile or semi-volatile solid and liquid samples.^4,5^ A small amount of sample is introduced to the ion source on the surface of a glass capillary, which forms the tip of the analysis probe. The sample is vapourised by a stream of hot nitrogen gas and ionised in a corona discharge before the ions are extracted from the ion source into the mass analyser. Mass spectrometers employing ASAP ion sources are commercially available from a number of manufacturers, usually in combination with a quadrupole or time-of-flight mass analyser.^6,7^ These instruments have found a wide variety of applications, including detection of opium alkaloids in poppy seeds,^8^ determination of drug purity,^9^ measurement of secondary organic atmospheric aerosols,^10^ and detection of *p*-phenylenediamine (PPD) in henna products.^11^ While clinical applications remain largely unexplored, given the capability of ASAP-MS to offer rapid profiling of metabolites present in biological samples, the technique holds significant promise in areas such as intraoperative diagnostics, disease screening, and pathology more generally.

Despite its promise for clinical usage, obtaining high quality and trustworthy data sets in ASAP-MS remains a challenge, often due to lack of a carefully standardised sample handling and measurement procedure. Considering the issue more broadly, in a survey involving 1576 scientists,^12^ over 70% disclosed difficulties in reproducing others’ experiments, and more than half faced challenges in repeating their own experiments.‡‡‘Repeatability' in a measurement is achieved when the measurement consistently produces the same outcomes under identical circumstances (same operator, apparatus, laboratory, and within a short period of time), while ‘reproducibility’ is defined as close agreement between measurements performed using the same method and identical test material but under different circumstances (*e.g.* different operators, apparatus, laboratories, or after a significant interval of time).^13,14^ Almost 90% of chemists participating admitted to experiencing failures in experiment replication. While not directly related to ASAP-MS, this study highlights the critical need for standardisation and careful experimental design. In the context of ASAP-MS this requires careful investigation and optimisation of a number of key experimental parameters, in order to enable the generation of trustworthy and reproducible clinical data sets, thereby enhancing the reliability of ASAP-MS for clinical applications.

In a previous study, we addressed standardisation issues in the measurement of human plasma using ASAP-MS.^15^ The present study builds on this earlier work to further optimise the methodology for generating meaningful and trustworthy clinical data sets, in this case on samples of human brain and cerebrospinal fluid. We investigate the influence on the mass spectra of calibration and post-calibration procedures, including the presence of residual calibration mix within the ion source;^16^ cooling of the probe tip following recording of background spectra; probe cleaning procedures; contamination from consumables such as lens tissue and sample storage containers;^17^ and variation between different instrument users. Lack of standardisation in any of these can lead to variations in the data in the form of contaminant peaks, changes in ionisation probability for some or all sample components, or sample degradation. If these variations are interpreted as significant features in classification models the result is significant skewing of results.

As part of the study, we also consider batch effects in some detail. As the name suggests, batch effects arise when samples are measured in different ‘batches’, resulting in systematic differences between subsets of data within a large data set.^18–20^ These have become more important in the age of big data.^21^ Some of the inter-batch differences may be reasonably easy to explain (*e.g.* batches of data from different labs or recorded using different instruments or different experimental protocols, or subjected to different data processing protocols). However, even if all experimental parameters are standardised as comprehensively as possible, some batch effects will usually remain. If these are not considered carefully and corrected for as far as possible, they can mask – or worse, mimic – biological variation, leading to highly misleading results. The conclusions of more than one study have been found to be invalid due to improper treatment of such effects; for example, Mertens *et al.*^20^ highlighted a number of clinical mass spectrometry studies in which poorly designed experiments resulted in the perfect confounding impacts of batch variation and biological variation. Although these experiments produced high accuracy rates in machine learning training, the affected data sets and conclusions were ultimately considered useless and abandoned. In the present study, alongside standardising the experimental protocol as far as possible, we evaluate several different methods for batch effect correction, and are able to make a series of recommendations in relation to optimised use of ASAP-MS for clinical data set generation.

## 2 Experimental

The frozen post-mortem brain and cerebrospinal fluid (CSF) samples used in this study were provided by the Oxford Brain Bank; a research ethics committee (REC) approved and Human Tissue Authority (HTA)-regulated research tissue bank (REC reference 15/SC/0639, issued by the NHS Health Research Authority ‘South-Central – Oxford C’). Upon collection of whole post-mortem brains by the Oxford Brain Bank, brains were dissected fresh. If the dura was present, it was removed from the cerebrum, and an incision was made along the longitudinal fissure to separate the cerebral hemispheres. The cerebellum was detached from the cerebrum at the level of the fourth ventricle and the brain stem were removed. The cerebellum was divided along the posterior Cerebellar notch, and one of the Cerebellar hemispheres was dissected into 1 cm thick slabs. The brain was then sectioned into 1 cm thick coronal sections starting at the level of the mammillary bodies. Whole hemisphere slabs were snap-frozen in liquid nitrogen vapour and stored at −80 °C until further dissection on dry ice, whereby the anterior cingulate gyrus was dissected at the level of the genu of the corpus callosum.

Samples were prepared for analysis as follows:

1. Brain samples were acclimatised to −20 °C and mounted onto a cryostat block using optimal cutting temperature (OCT) compound, ensuring that cut sections were not contaminated with OCT. Three 10 μm sections were obtained from each sample, and transferred to a polypropylene sample tube (see later for details of the various tubes employed). To create a suitable medium for homogenisation, 100 μL of LC-MS grade water (Fisher) was added to the tube. A bead homogeniser (OMNI International bead ruptor elite) was then employed to thoroughly homogenise the sample (see Table S1 of the ESI† for homogeniser settings).

2. CSF samples were thawed from −80 °C and centrifuged at 12 000*g* for 15 minutes at 4 °C to separate the cellular components and particulate matter from the liquid fraction. The supernatant was then pipetted into a new tube, taking care not to disturb the pellet.

All measurements were made on an Advion Expression version L compact quadrupole mass spectrometer equipped with an ASAP ion source and controlled by Advion Mass Express data acquisition software (version 6.9.38.1). The ion source was run in ‘high temperature, low fragmentation’ positive ion mode – see Table S2 of the ESI† for detailed instrument settings. Prior to measurements, the glass capillaries that comprise the tip of the ASAP probe were baked in an oven at 250 °C for 30 minutes to remove any surface contaminants as far as possible. To make a measurement, the probe was fitted with a clean glass capillary and inserted into the ion source for 30 s to record a background mass spectrum. The probe was then removed from the ion source and allowed to cool before being brought into contact with a small amount of sample and reinserted into the ion source for a measurement time of 25 s. Due to the high sensitivity of the instrument, in general the smallest possible amount of sample should be transferred to the probe tip for measurement. When the probe is inserted into the ion source this should result in an almost instantaneous rise in the total ion signal, followed by a rapid decay over the next 20–30 s. Too much sample leads to signal saturation, characterised by high signals that do not decay or strange time-dependent behaviour of the total ion signal. Examples can be found in a previous publication,^15^ in which we characterised this behaviour in detail.

Mass spectra were exported for analysis using Advion Data Express data manipulation software (version 6.9.38.1) and processed using custom Python software (Jupyter Notebook, available at https://github.com/Liwensong2019/ASAP-MS). The mass spectrometer completes a scan every 900 ms during the acquisition period, and the raw data files contain all of these scans stored in sequence. Some of these scans correspond to ‘background’ recorded when the probe was not present in the ion source, and some correspond to signal recorded during each of the 30 s intervals when the probe loaded with sample was inserted into the ion source. The Python script identifies these regions based on the rapid rise in the total ion count on each insertion of the probe, and calculates the average and standard deviation of the mass spectra recorded over each 30 s sample measurement. The script also generates a background spectrum in an equivalent way from the 30 s background measurement, and subtracts this from each of the ‘signal’ measurements. The resulting background-corrected spectra for each sample are then normalised to unit area under the spectrum, and averaged to generate a single mass spectrum for each sample.

The effects of a number of experimental factors were investigated:

1. Calibrant and background effects: the mass spectrometer is calibrated daily with Advion APCI calibration tuning mix in order to ensure repeatable peak positions and widths in the mass spectra. A considerable amount of tuning mix is injected through a capillary tube inlet into the ion source during the calibration process, and any residual mix can have a significant effect on both the ‘background’ and ‘sample’ mass spectra recorded subsequently. Fig. S1† shows examples of background spectra recorded in the presence and absence of residual tuning mix. To assess the impact of residual tuning mix on clinical sample data sets, a CSF sample prepared as detailed above was split into two. Each of the two sub-samples were subjected to 25 repeat ASAP-MS measurements, with the measurements on one sub-sample performed immediately after calibration when residual tuning mix was present, and those on the second sub-sample performed after allowing the instrument to run without sample introduction until a clean and stable background was observed. After analysing the repeatability of the two groups of measurements, two methods for removal of residual tuning mix from the ion source were investigated. These involved flowing either (i) air or (ii) a 1 : 1 mix of LC-MS water and ethanol through the capillary inlet for 1.5 minutes, then running the instrument for 8.5 minutes to clear the ion source and allow the background signal to stabilise prior to making any measurements.

2. Temperature of ASAP probe tip: after each background measurement, during which the glass capillary that forms the tip of the ASAP probe is exposed to nitrogen gas at 400 °C, a period of cooling is required prior to sample loading and measurement. Cooling curves were measured using a thermal imaging camera (FLIR C3-X Compact Thermal Camera) immediately after removing the probe from the ion source (see Fig. S2 of the ESI† for a schematic of the experimental setup, and Table S3† for camera settings). The highest temperature region of the image was identified by the thermal camera's ‘hot spot’ function, and was used to define the measurement region corresponding to the probe tip. The temperature in the measurement region was recorded every second during imaging of the probe, and the results from ten repeat measurements were used to generate a cooling curve. The room temperature was 23 °C on the day of the measurement.

3. Glass capillary cleaning/reuse: in the interests of cost reduction and sustainability, we have established in previous work^15^ that with appropriate cleaning the glass capillary forming the tip of the ASAP probe can be reused for up to five measurements on the same sample without any negative impact on the measured mass spectra. Between measurements, the capillary is cleaned by rinsing with deionised water, followed by gentle wiping and drying with lens tissue. In the present work, we investigated whether the performance could be improved further by inserting the cleaned capillary into the ASAP source in order to expose it to the hot nitrogen gas flow, followed by removal and cooling for 20 s. The comparison was performed by analysing one frozen brain sample 25 times with the two different cleaning protocols, recording five repeats with five different capillaries for each protocol.

4. Consumables: to investigate the impact of different consumables on the mass spectra, we selected three different brands of polypropylene tubes (1.5 mL polypropylene tubes (Tube Brand 1, PCR clean, manufactured without slip agents, plasticisers, and biocides) were purchased from Eppendorf; 2 mL polypropylene tubes (Tube Brand 2, DNase- and RNase-free) were obtained from OMNI; 10 mL optically clear polypropylene tubes (Tube Brand 3, DNase-, RNase-, Endotoxin-, and Pyrogen-free) were sourced from Appleton) and three different brands of lens tissues (MC-5 Lens Tissues (Lens Tissue Brand 1) were purchased from THORLABS; additional lens tissues were obtained from KimTec (Lens Tissue Brand 2) and Fisher, UK (Lens Tissue Brand 3)). To investigate contaminant peaks in the mass spectra arising from the polypropylene tubes, we first used moderate force to swab the inner wall of the empty tubes with the glass capillary tip of the ASAP probe, and recorded mass spectra. To investigate any diffusion of materials from the tube to the solvent, we added LC-MS water to the tubes and left them overnight, before recording mass spectra of the resulting solutions. To investigate contaminant peaks arising from the lens tissues, we mimicked a standard cleaning procedure by gently wiping the glass capillary ASAP tip with each brand of lens tissue and then recorded mass spectra. To ensure consistent results, the measurements on each consumable were repeated five times.

5. Measurement repeatability between users: in order to investigate variations between different instrument operators in mass spectra recorded using a standardised protocol, we selected four representative users with varying scientific backgrounds and different levels of experience with ASAP-MS measurements. The four users made independent measurements on the same human cerebellum sample on the same day in a random order. We assessed both the repeatability within each individual user's measurements and the reproducibility across all four users.

### 2.1 Data analysis

Principal component analysis was used to investigate the repeatability and reproducibility of measurements made under the various different conditions investigated. For each data set, the individual measurements were reduced to their first five principal components. To visualise the data, the first two principal components (PC1 and PC2), which together account for around 85% of the variance in the mass spectra were plotted, together with a ‘confidence ellipse’ of one standard deviation (*i.e.* the ellipse defines a region within which 68% of the data points are expected to lie, assuming the data follow an approximately normal distribution). Prior to this, we performed a Shapiro–Wilk test to establish the normality of the data^22^ (see Table S4 of the ESI† for details). The resulting plots enable a comparative analysis between groups of measurements. The centroid or mean of each group was calculated by averaging each principal component across all measurements within the group, while the variability within each group was quantified by finding the standard deviation of the Euclidean distances between each measurement and the centroid.

The significance of any similarities and differences between the measurement groups was evaluated using various statistical methods. Since several of the data sets did not follow strictly normal distributions, for single-pair comparisons, we employed the Mann–Whitney *U* test^23^ within the SciPy (version 1.5.4) Python package^24^ (this package was also used for the Shapiro–Wilk test mentioned above). For multiple group comparisons, we used a two-step approach: first a one-way Analysis of Variance (ANOVA) to determine overall differences among groups, followed by Tukey's Honestly Significant Difference (HSD) test for pairwise comparisons.^25^ These tests were implemented using the Python statmodels package (version 0.13.5).^26^ See Table S4 of the ESI† for further details. Significance levels were set at *p* < 0.05, *p* < 0.01, and *p* < 0.001 for all tests.

### 2.2 Batch effects

Batch effects were investigated using frozen brain samples from the cerebellum and anterior cingulate cortex regions of six patients, *i.e.* 12 samples in total. Each sample was split into two, and used to generate two data sets as follows:

1. All 12 samples were measured within a single day, generating a data set that serves as a control without batch effects;

2. The 12 samples were measured in two batches on two different days, generating a data set with the potential presence of batch effects. The sample distribution between the two batches was randomised in order to mitigate any bias, with each batch including samples from both brain regions of three different patients.

To examine the batch effects, PCA plots were generated for all the data points in each batch, with confidence circles centred at the centroid and extending to the furthest data point in each batch. The function Kruskal from SciPy (version 1.5.4) was used to perform a Kruskal–Wallis (kW) *H*-test in order to evaluate the statistical significance of correlations between the principal components (PCs) and the batches.^24,27^ This dual approach enabled us to rigorously investigate any potential batch-related discrepancies, ensuring the reliability of our findings. Having identified batch effects, we explored the efficacy of correcting for these using two different batch-effect correction tools available in Python, namely ComBat and Independent Component Analysis (ICA).^18,28,29^

## 3 Results and discussion

### 3.1 Calibrant and background effects

**Fig. 1:**
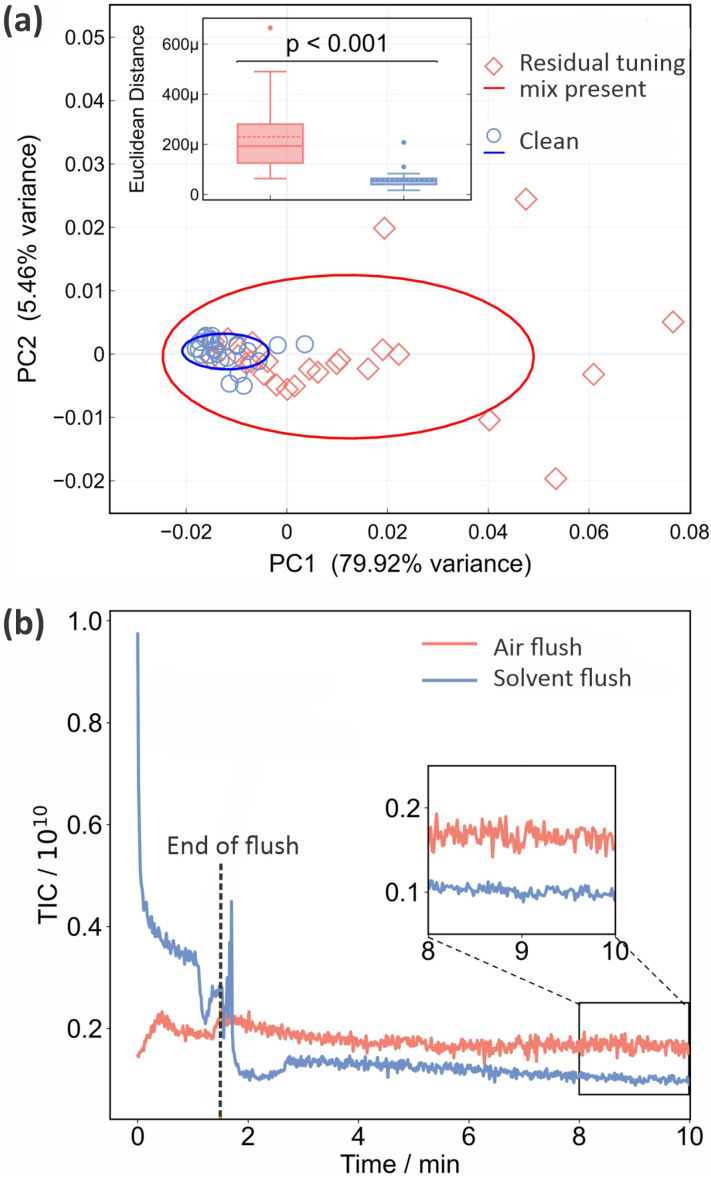
The effect of residual calibration tuning mix in the ion source on the repeatability of ASAP-MS measurements: (a) PCA plot (see Section 2.1 for detailed description) of 25 repeat measurements on the same CSF sample with (red diamonds) and without (blue circles) residual tuning mix present in the ion source. The inset shows the results of a Euclidean distance *t*-test comparison between the two conditions, demonstrating a significant difference; (b) comparison of the total ion signals (total ion count, TIC) recorded as a function of time when the ion source is flushed with air (red line) or a 1 : 1 mixture of LC-MS water and ethanol ASAP-MS. The ion source is flushed for the first 1.5 minutes, before the inlet valve is closed and the signal allowed to stabilise over the following 8.5 minutes. *p* values are from a Mann–Whitney *U* test.

As noted in Section 2, residual tuning mix in the ion source following spectrometer calibration can have a significant effect on both the background and sample mass spectra recorded subsequently. This is shown quantitatively in Fig. 1. Panel (a) shows PCA plots for the two sets of 25 mass spectra recorded for a sample of CSF immediately after calibration, and after running the spectrometer until no tuning mix remained in the ion source, respectively. The corresponding mass spectra and associated information are shown in Fig. S3–S5 of the ESI.† We see from Fig. 1 that the data recorded under ‘clean’ conditions, shown in blue, are tightly clustered and highly reproducible, while those recorded with residual tuning mix present, shown in red, are scattered over a large area of PCA space, showing a much higher degree of variation. A *t*-test performed on the Euclidean distances of the principal component vectors from the centroid for each data set (see Section 2.1) shows a significant difference between the two sets of measurements, with *p* < 0.001. The variability in the mass spectra recorded in the presence of residual tuning mix is most probably caused by a combination of time-varying contributions to the mass spectra from the tuning mix ‘contaminant’ peaks and ion suppression effects that alter the contributions from sample peaks.

Unsurprisingly, we can conclude that ensuring a clean background and removing contaminant *m*/*z* peaks (from any source) as far as possible yields improved measurement repeatability. Before moving on to consider active methods for eradicating signals from residual tuning mix prior, we note that frequent cleaning of the ASAP ion source is an important measure that should be taken in order to minimise background interference and enhance data quality. The required cleaning frequency is sample-dependent, but as an example, when processing large numbers of plasma samples in our own laboratory we clean the source after around 150 measurements.

As explained in Section 2, we investigated two different flushing methods for removing residual tuning mix from the ASAP ion source, employing air or a 50 : 50 mix of ethanol and LC-MS water, respectively. The results are shown in Fig. 1(b) in the form of plots of total ion count (*i.e.* the integrated signal across the entire mass spectrum) as a function of time. Air (red line) or solvent (blue line) is flushed through the ion source for the first 90 seconds, before the inlet valve is closed and signal is recorded for a further 8.5 minutes. Compared with the air flush, flushing with solvent results in much higher signal levels during the flush, but considerably lower and more stable signal levels (see inset to figure) once equilibrium is reached following the flush. As well as reducing the overall ion count more effectively, the solvent flush was also more effective at removing the specific *m*/*z* peaks arising from the tuning mix (see Fig. S5 of the ESI†), and was the flushing method of choice for all subsequent measurements.

### 3.2 Temperature of ASAP probe tip

**Fig. 2:**
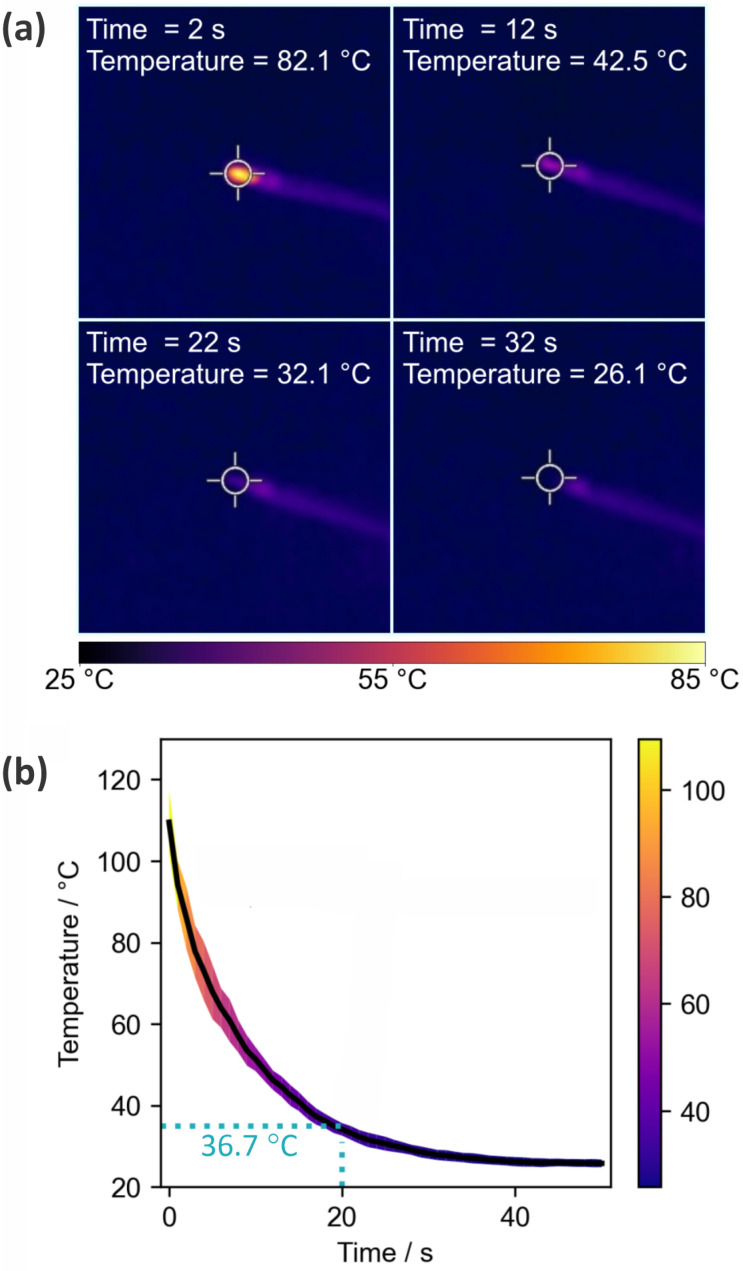
(a) Thermal images of the ASAP probe tip recorded 2 s, 12 s, 22 s, and 32 s after removal of the probe from the mass spectrometer ion source; (b) cooling curve for the probe tip following removal from the ion source, averaged over ten measurements. The solid line is the mean, with the shaded area indicating the standard deviation of the ten measurements.

Fig. 2 shows the results of the thermal imaging experiments used to record cooling curves for the ASAP probe after removal from the ion source. The probe is simply left to stand under ambient conditions in the laboratory during the cooling period. Panel (a) shows thermal images of the probe at four different time points, while panel (b) shows the temperature as a function of time.

The cooling curve shows the expected exponential decay, with rapid cooling to a little over 100 °C occurring during the few seconds taken to remove the probe from the ion source and position it in front of the camera. The tip reaches a temperature of ∼37 °C after around 20 s, after which it is cool enough for loading of biological samples. After 30 s the tip has cooled to 26 °C, just a little over room temperature.

### 3.3 Glass capillary cleaning/reuse

**Fig. 3:**
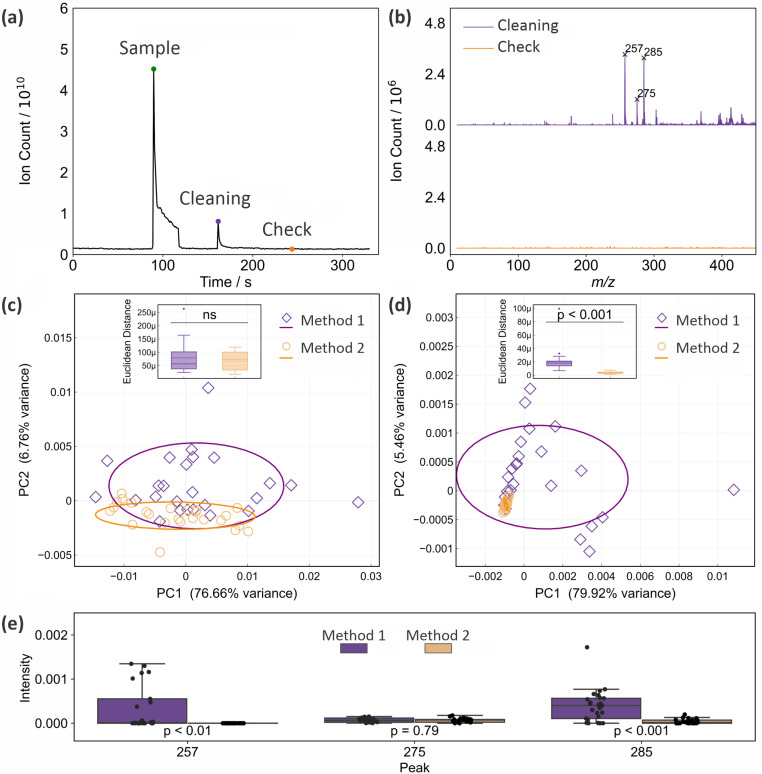
Evaluation of two different cleaning protocols for the glass capillary tip of the ASAP probe: (a) total ion count recorded as a function of time during a sample measurement, cleaning step, and ‘check’ measurement for cleaning Method 2; (b) mass spectra recorded during the ‘cleaning’ and ‘check’ insertions of the ASAP probe; (c) PCA plots for 25 mass spectra (full *m*/*z* 10–1000 range) of a frozen brain sample recorded using cleaning methods 1 and 2; (d) as for (c), but considering only the mass range from *m*/*z* 200–300; (e) Box and whisker plots comparing the intensities of three mass peaks arising from contaminants associated with the lens tissue used for cleaning at *m*/*z* 257, 275, and 285 when cleaning methods 1 and 2 are employed. *p* values are from a Mann–Whitney *U* test.

As noted in Section 2, we have established previously^15^ that with appropriate cleaning between measurements, the glass capillary tip of the ASAP probe can be reused up to five times before it should be replaced with a clean capillary. In the previous work, the tip was cleaned with deionised water and lens tissue between uses (‘Method 1’). In the present work we investigated whether performance can be improved further by including an additional cleaning step after the manual cleaning, in which the clean probe is inserted into the ion source and exposed to the hot nitrogen flow for 25 s before being removed, allowed to cool for 20 s, and used for the next measurement (‘Method 2’). Fig. 3(a) shows the total ion count recorded as a function of time during a sample measurement, during the period when the probe is reinserted after manual cleaning and exposed to the hot nitrogen flow, and when the probe is reinserted again to check the effectiveness of the cleaning protocol. The mass spectra recorded for the last two (‘cleaning’ and ‘check’) measurements are shown in Fig. 3(b). We see that there is still significant ion signal during the time when the probe is inserted into the ion source for cleaning, with significant peaks that can be attributed to residue from the lens tissue used in the manual cleaning step (see Section 3.4). This residual signal disappears following the two cleaning steps.

Fig. 3(c) and (d) show PCA plots for the two sets of 25 mass spectra recorded for a frozen brain sample using cleaning methods 1 and 2 (the complete set of mass spectra used in this analysis can be found in Fig. S6 and S7 of the ESI†). Panel (c) shows principal components for the full *m*/*z* 10–1000 mass range of the mass spectrometer, while panel (d) shows the results for a truncated mass range of *m*/*z* 200–300, chosen to isolate the contributions from mass peaks associated with the lens tissue used in the manual cleaning step. In both cases the spread in the data is reduced considerably when using cleaning method 2. Despite the clear reduction of spread in the PCA plot, a *t* test shows that this reduction is not statistically significant when the complete mass spectra are considered; however, it is highly significant (*p* < 0.001) over the *m*/*z* range in which the lens tissue peaks appear. These results suggest that while it can be difficult to detect significant influences of contaminants on the complete mass spectra, explaining why such contamination can sometimes be accidentally and subtly introduced into clinical data sets, local influences can be highly significant, and should be minimised wherever possible.

Fig. 3(d) shows box and whisker plots of the intensities recorded using both cleaning methods for the three most intense peaks arising from lens tissue residue. Significant reductions are seen in the intensities of the peaks at 257 and 285, while the peak at *m*/*z* 275 is sufficiently low in intensity under all conditions that the second step does not lead to a statistically significant reduction. Further analysis of these data can be found in Fig. S8 of the ESI.†

We can conclude that cleaning method 2 is superior to cleaning method 1, and this method was employed in all subsequent measurements. This includes all measurements reported in this manuscript, unless stated otherwise.

### 3.4 Consumables

**Fig. 4:**
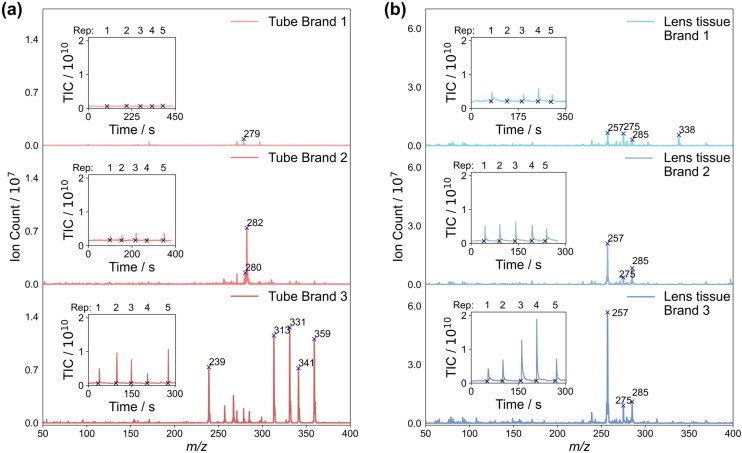
Mass spectra recorded for (a) swabs from the inner wall of three different brands of polypropylene tubes, and (b) three brands of lens tissue (see Section 2 for brand details), averaged over five measurements. The total ion count recorded during the five measurements is shown in the inset to each mass spectrum. Note the difference in intensity scales used to plot the two sets of mass spectra.

Consumables can add a variety of different contaminant peaks to the mass spectra, which we investigated by performing ASAP-MS measurements on three different types of sample storage tubes, and three different types of lens tissue. Details of the products used can be found in Section 2. Mass spectra recorded by swabbing the sample tubes and lens tissues with the ASAP probe, averaged over five repeat measurements, are shown in Fig. 4(a) and (b), respectively. The total ion counts recorded during the five measurements are shown as insets to each mass spectrum. For both the storage tubes and lens tissue, we see large differences in the mass spectra recorded for the three different brands.

Considering first the sample tubes, brand 1 yields consistently low total ion signals and a small number of very low intensity peaks in the mass spectrum, in line with previous work by Canez *et al.*^17^ In contrast, brands 2 and 3 yield significantly higher total ion signals and – particularly in the case of brand 3 – a large number of high-intensity peaks in their mass spectra. A second set of measurements, made on LC-MS water left overnight in each brand of tube (see Fig. S9 of the ESI†), yielded very low signal for all three brands of tube. This suggests that all three tubes are generally suitable for use with ASAP-MS analysis as long as direct contact of the ASAP probe with the tube walls is avoided.§§Note that all samples were homogenised in water. Other solvents were not tested, and in principle may yield different results.

Considering next the three brands of lens tissue, we see large total ion signals and numerous peaks in the mass spectra for all three brands. In all three cases, three of the most intense peaks appear at *m*/*z* 257, 275, and 285, with brand 1 yielding another peak of high intensity at *m*/*z* 338. While these signals have the potential to contaminate the sample mass spectra, as shown in Section 3.3, residue from lens tissue used to clean the ASAP probe tip can be removed effectively simply by inserting the probe into the hot N_2_ gas flow inside the mass spectrometer ion source for a short period of time prior to making measurements on samples.

We can conclude that careful and consistent selection of consumables, and clear and consistent handling protocols, are likely to be key in order to avoid the potential for brand-specific contamination of the sample mass spectra. New consumables should be evaluated carefully in order to establish their potential to contaminate mass spectra, either directly or *via* ion suppression effects. Direct contact of the sample or probe with the consumables should be avoided where possible, and rigorous cleaning techniques should be employed when reusing glass capillaries in the ASAP probe. Caution should also be exercised in situations where *m*/*z* peaks arising from consumables overlap with ‘biological’ peaks arising from the sample. Ideally, such peaks should not be used for classification, and in cases where this is not possible, interferences should be carefully considered and quantified.

### 3.5 Measurement reproducibility between users

**Fig. 5:**
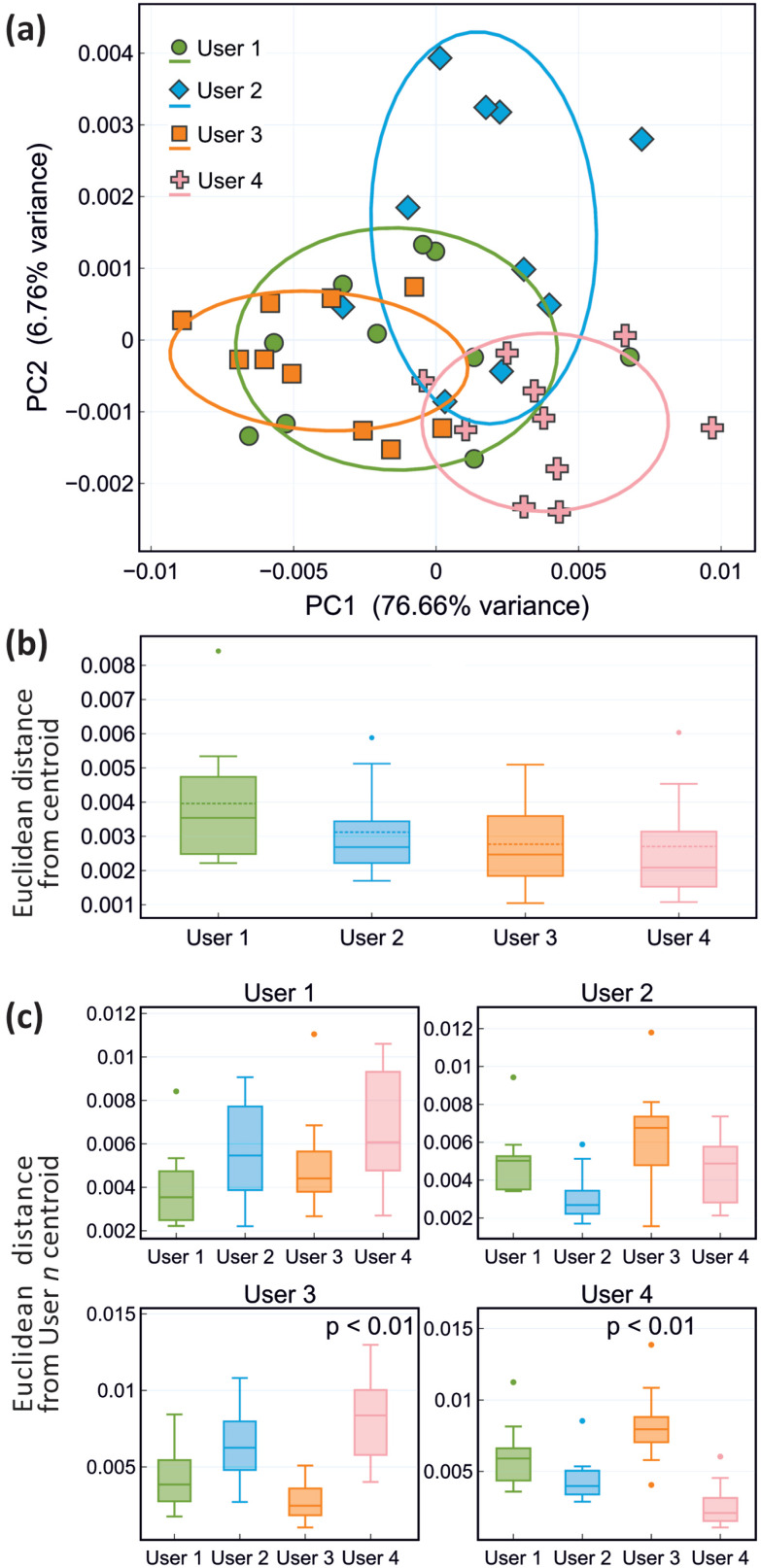
Evaluation of measurement repeatability for a single sample of cerebellum between four different users: (a) PCA plot, with confidence ellipses represent one standard deviation for each user; (b) Box and whisker plot of the repeatability achieved by each user, determined from the Euclidean distances of each user's measurements from their own measurement centroid; (c) As for (b), but comparing each user's measurements with the centroids of other users. Statistically significant deviations are labelled with their *p* value, determined from a Mann–Whitney *U* test.

Fig. 5 shows PCA plots and the corresponding reproducibility analysis for ten repeat measurements on a frozen cerebellum sample made by four different users. Based on the results shown in Fig. 5(b), there are no significant differences in the repeatability achieved by each of the four users, with mean deviations of each users measurements from their own centroid of between 0.002 and 0.0035; each user is able to measure the sample consistently, with minimal variability within their own data sets. However, when the results obtained by different users are compared with each other, we see more significant differences, with mean deviations from the centroid of other users of up to 0.006. These results suggest that despite consistent performance of individual users, variations between users occur even when the same protocol is followed rigorously. In the context of a metabolomics or other clinical study, it is therefore important to evaluate user reproducibility carefully and quantitatively to ensure that any variability in the data introduced by multiple users is (significantly) smaller than the biological variation under study.

## 4 Batch effects

As noted in Section 1, when acquiring large clinical data sets, variations in the mass spectra recorded for different batches of samples can be very significant in comparison with the biological variations under study. The reasons for these variations are not well understood, but any study of this type needs to take batch effects into account and implement measures to mitigate or remedy them.

**Fig. 6:**
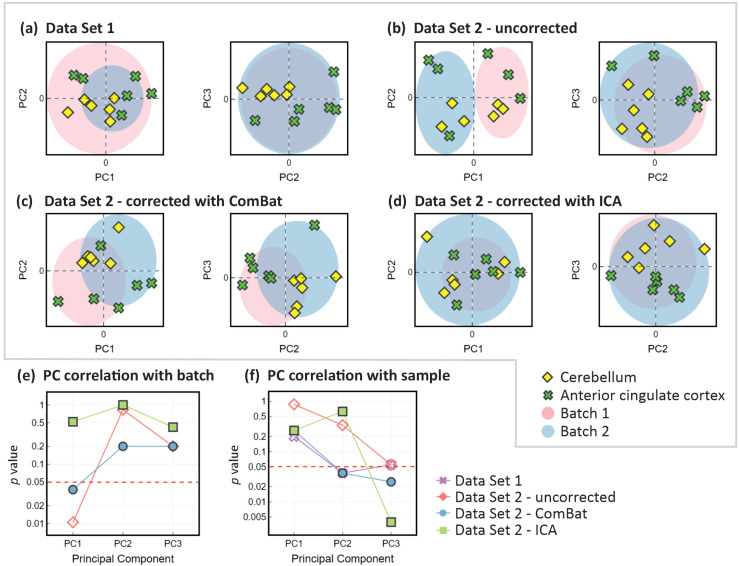
Batch effects and mitigation through the use of batch effect correction methods. Panels (a) and (b) show PCA plots (PC1 *vs.* PC2 and PC2 *vs.* PC3) with one-standard-deviation confidence ellipses (shaded regions) for Data Set 1 (recorded in a single batch) and Data Set 2 (recorded in two batches on separate days), respectively. Panels (c) and (d) show the analogous PCA plots for Data Set 2 after correction using the ComBat and ICA methods, respectively. Correlations (in the form of *p* values) between the first three principal components of the mass spectra and (e) batch and (f) sample type, for Data Set 1 and Data Set 2 before and after batch correction. The red dashed line indicates the upper threshold for the region of statistical significance, with *p* < 0.05 (Mann–Whitney *U* test).

The problem is illustrated quite clearly even using the small number of measurements employed in the present study. As explained in Section 2, we recorded spectra from 12 brain tissue samples from six patients, with cerebellum and anterior cingulate cortex samples for each patient. The patients were randomised into two different groups, with their samples comprising ‘batch 1’ and ‘batch 2’, respectively. To generate Data Set 1, which we can use as a control, measurements were made on all 12 samples (both batches) on the same day, while for Data Set 2 the two batches were analysed on different days. Fig. 6(a) and (b) show PCA plots (PC1 *vs.* PC2 and PC2 *vs.* PC3 in each case) for the two data sets, with the centroid rings superimposed as shaded regions in each case. For Data Set 1 (Fig. 6(a)), we see good overlap between the measurements made on the two batches, implying that they were recorded under consistent measurement conditions. For Data Set 2 (Fig. 6(b)) we see significant separation between the two sets of measurements, particularly in the first principal component, and more overlap between data points for the two different tissue types. To the best of our ability, the measurements were all made under identical conditions, so this is a clear example of a batch effect, in line with those observed by other authors.^19^ If not corrected or appropriately accounted for, the batch effect could easily confound any effects arising from true biological variability, and lead to the drawing of highly misleading conclusions. Some further analysis of the mass spectral features that contribute most significantly to the batch effects can be found in the ESI.†

In Fig. 6(c) and (d), we show the results of performing a batch correction on Data Set 2, using the ComBat and independent component analysis (ICA) methods, respectively. The ComBat algorithm^18,28^ assumes that batch effects affect many *m*/*z* peaks in similar ways and uses an empirical Bayesian approach to adjust for these effects. In the ICA method,^29^ the complete data set is factorised into components using one of a number of matrix factorisation methods, and the components that show significant correlation with the individual batches are removed. Inspecting the data in Fig. 6(c) and (d), we see that both methods yield a reduction in the batch-related separation along PC1 and improve the clustering according to sample type (cerebellum vs anterior cingulate cortex). However, the data corrected using the ComBat method still show separation by batch, while the separation is almost completely removed by the ICA method. The latter results in almost complete overlap of the centroid rings, similar to that observed in Data Set 1, which was recorded in a single batch. In the present example, the ICA method appears to perform better than the ComBat method, but this would need to be tested much more extensively with larger data sets before drawing any firm conclusions on this point.

Fig. 6(e) and (f) show the results of a more in-depth statistical analysis of the batch effect correction, in the form of a correlation analysis. Panel (e) shows *p* values for the correlation between batch and the first three principal components of Data Set 2 before and after batch correction using the ComBat and ICA methods. Before batch correction (red diamonds), the data show a statistically significant correlation with batch (in the plot they lie below the red dashed line indicating *p* < 0.05). Batch correction with the ComBat method (blue circles) reduces the correlation, but *p* still indicates a significant correlation, while the ICA method reduces the correlation substantially (*p* ∼0.5). Panel (f) explores the correlation between the principal components of the data sets and the sample type for Data Set 1 and for Data Set 2 before and after batch correction. For Data Set 1, recorded in a single batch, PC2 (and perhaps PC3) reveals a statistically significant correlation with sample type. For Data Set 2, PC2 shows no significant correlation with sample type before batch correction. The correlation becomes significant following correction with the ComBat algorithm, but (interestingly given the apparent overall better performance of this algorithm) not with the ICA algorithm. Correlation between sample type and PC3 becomes statistically significant for Data Set 2 following correction with either approach. These findings suggest that while batch effects can obscure true biological variation, employing batch effect correction methods can recover these biological differences to varying degrees. However, as noted by other authors,^30^ it is often not possible to remove batch effects completely.

Based on our small proof-of-concept demonstration, we can conclude cautiously that batch effects are significant in ASAP-MS measurements, but that they can be mitigated by the use of batch effect correction methods. However, further investigation with much larger data sets is needed before drawing any firm or quantitative conclusions on the most effective correction methods or the extent to which biological variations can be recovered.

## 5 Conclusions

We have investigated a number of factors that can degrade the quality of clinical data sets based on ASAP-MS measurements of tissue or fluid samples, and have identified ways to mitigate these effects. Based on the current investigation, and our previous work on plasma,^15^ we recommend:

(i) Ensuring utmost cleanliness of the ion source, which should be free from all contaminants, including residual calibration tuning mix;

(ii) Ensuring sufficient cooling of the ASAP probe tip before loading with biological samples;

(iii) Developing an appropriate cleaning protocol for the probe between measurements. Standard cleaning approaches can be enhanced by an additional step in which the probe tip is exposed to the hot N_2_ gas flow inside the ASAP ion source for a short period of time;

(iv) Consumables must be carefully evaluated for their potential to introduce contaminant peaks to the mass spectra, and should be chosen to minimise such contamination;

(v) All measurement procedures should be standardised as far as possible *and written down clearly and comprehensively* so that they can be followed as closely as possible by different users;

(vi) Even with strict adherence to well-designed standard operating procedures, it is not possible to avoid batch effects arising from both known (*e.g.* different users) and unknown causes, and these should be carefully considered and corrected where possible. In the present study, we have shown using a small set of sample data that both the ComBat and ICA methods for batch correction can mitigate these effects to varying degrees and preserve true biological variations, opening the way to further, more quantitative studies with larger data sets.

As a relatively low-cost, rapid, and straightforward measurement technique, ASAP-MS holds considerable appeal for clinical applications. However, it is still a relatively new technique in the clinical arena, and measurement protocols must be carefully optimised in order to maximise its potential. The work presented in this paper and in our previous study on plasma measurements^15^ lays down some of the foundations for achieving these goals.

## Author contributions

LS: conceptualisation, methodology, investigation, software, data curation, formal analysis, visualisation, writing – original draft. JGR: methodology, investigation, validation, writing – review. MAP: investigation, writing – review and editing. CL: investigation. ASJEB: methodology, writing – review. BS: methodology. OA: funding acquisition, resources, supervision, writing – review. CV: project administration, methodology, funding acquisition, supervision, writing – review and editing, final draft.

## Data availability

Most of the data supporting this article have been included as part of the ESI.† Any data not included in the ESI† are available on request from the corresponding author.

## Conflicts of interest

The authors have no conflicts of interest to declare.

## Supplementary Material

AN-150-D5AN00166H-s001
