# Supplementary material for: Advancing atmospheric solids analysis probe mass spectrometry applications: a multifaceted approach to optimising clinical data set generation
Source: Analyst. 2025 May 12;150(12):2620–31. doi: 10.1039/d5an00166h (PMC12080459; doi:10.1039/d5an00166h)
Supplement: AN-150-D5AN00166H-s001 [file AN-150-D5AN00166H-s001.pdf]

Supplementary Information (SI) for Analyst.
This journal is © The Royal Society of Chemistry 2025

Supporting Information:

Advancing atmospheric solids analysis probe mass spectrometry applications: a multifaceted approach to optimising clinical data set generation

Liwen Song, Jasmine G. Reese, Michael A. Platt, Claire Lewis, Annabel S.J Eardley-Brunt, Bo Sun, Olaf Ansorge and Claire Vallance*

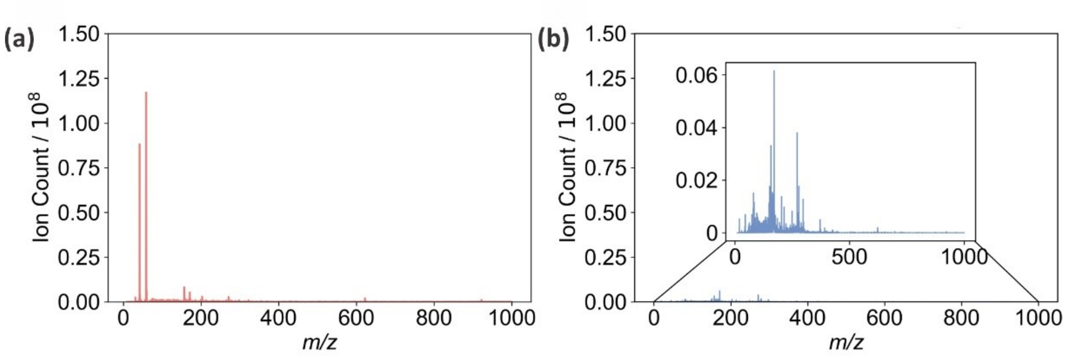

Figure S1: Examples of background mass spectra taken under different conditions in ASAP-MS: (a) a background spectrum taken when residual calibration tuning mix was present in the ion source; (b) an example of a clean background spectrum obtained after flushing the sample line with a 50:50 mixture of LC-MS ethanol and LC-MS water. The inset to (b) shows the low-mass peaks on a magnified scale.

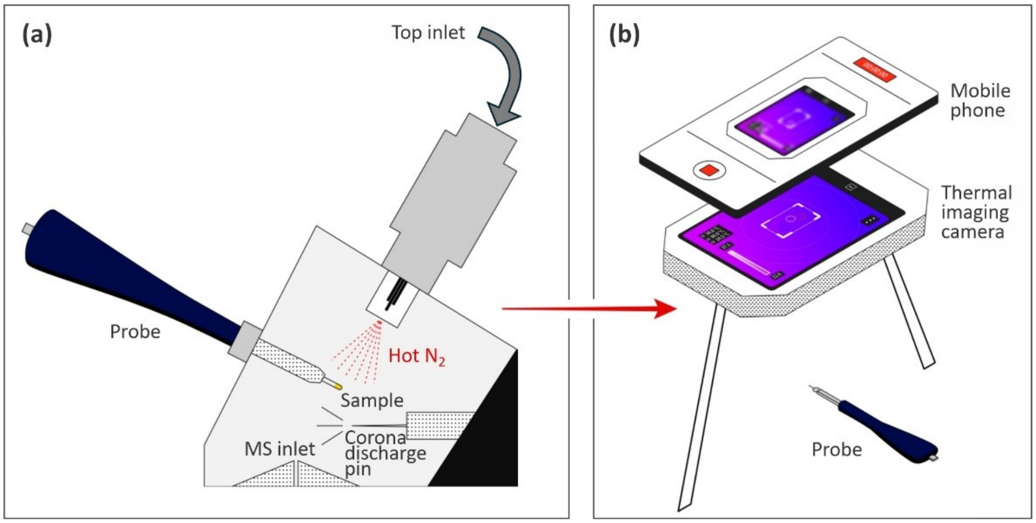

Figure S2: (a) Schematic of the ASAP-MS ion source, showing the inserted probe with glass capillary tip, hot N_2 gas flow, corona discharge, and inlet to the ion optics and mass analyser; (b) Setup used for thermal imaging of the ASAP probe tip. A region of interest in the image is defined, corresponding to the tip. Additional time-resolved imaging was achieved by using a mobile phone to capture video.

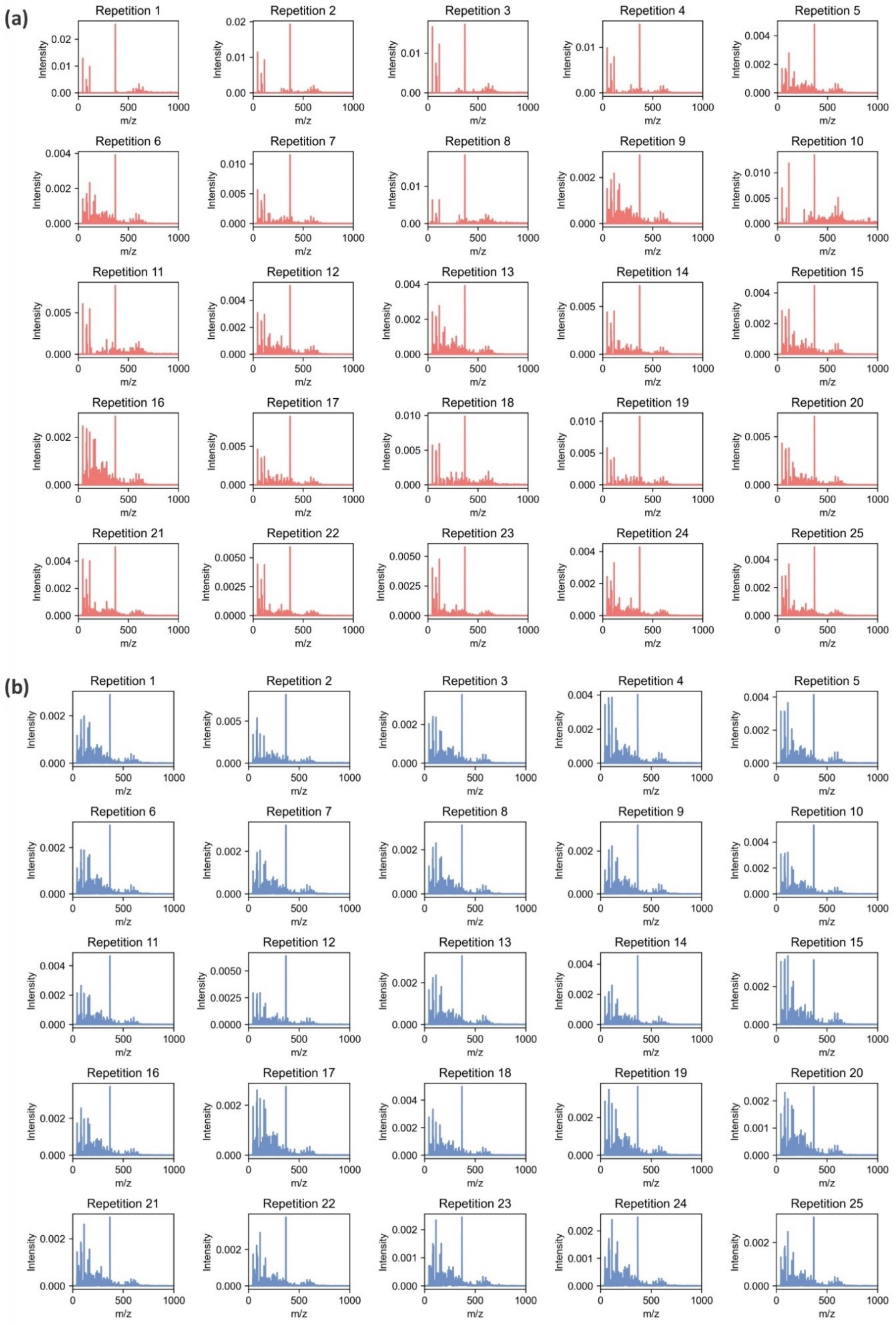

Figure S3: All mass spectra recorded for a CSF sample in the evaluation of the effect of residual background mass peaks on the repeatability of the measurements (see main text for details): (a) CSF sampled shortly after calibration, with residual tuning mix peaks present; (b) CSF sampled after running the spectrometer untl no tuning mix remained in the source, i.e. clean background.

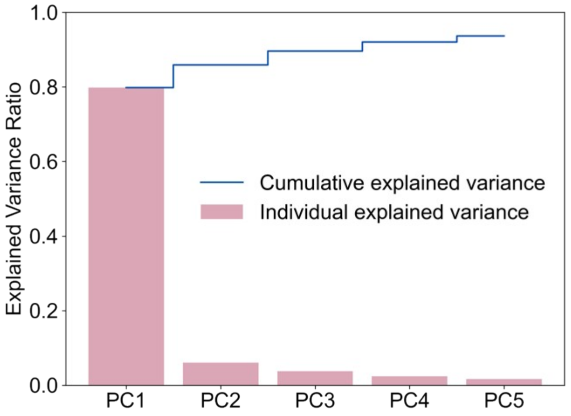

Figure S4: The cumulative explained variance plot from the PCA analysis of the mass spectra shown in Figure S3. The first five principal components (PCs) together explain over 93% of the total variance

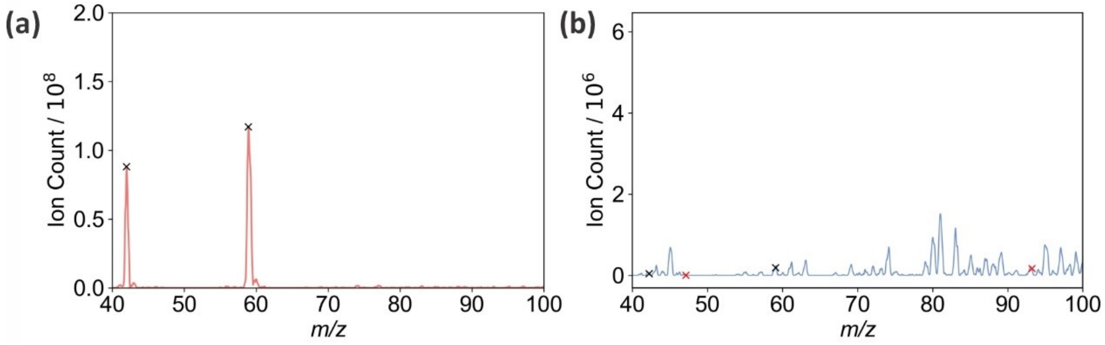

Figure S5: Examples of the low-mass region of mass spectra recorded under different background conditions (a) mass spectrum recorded with residual tuning mix present in the ion source; (b) mass spectrum recorded after running the spectrometer until no tuning mix remained in the ion source, i.e.clean background. Positions of peaks arising from residual tuning mix and from ethanol are marked with black and red crosses, respectively.

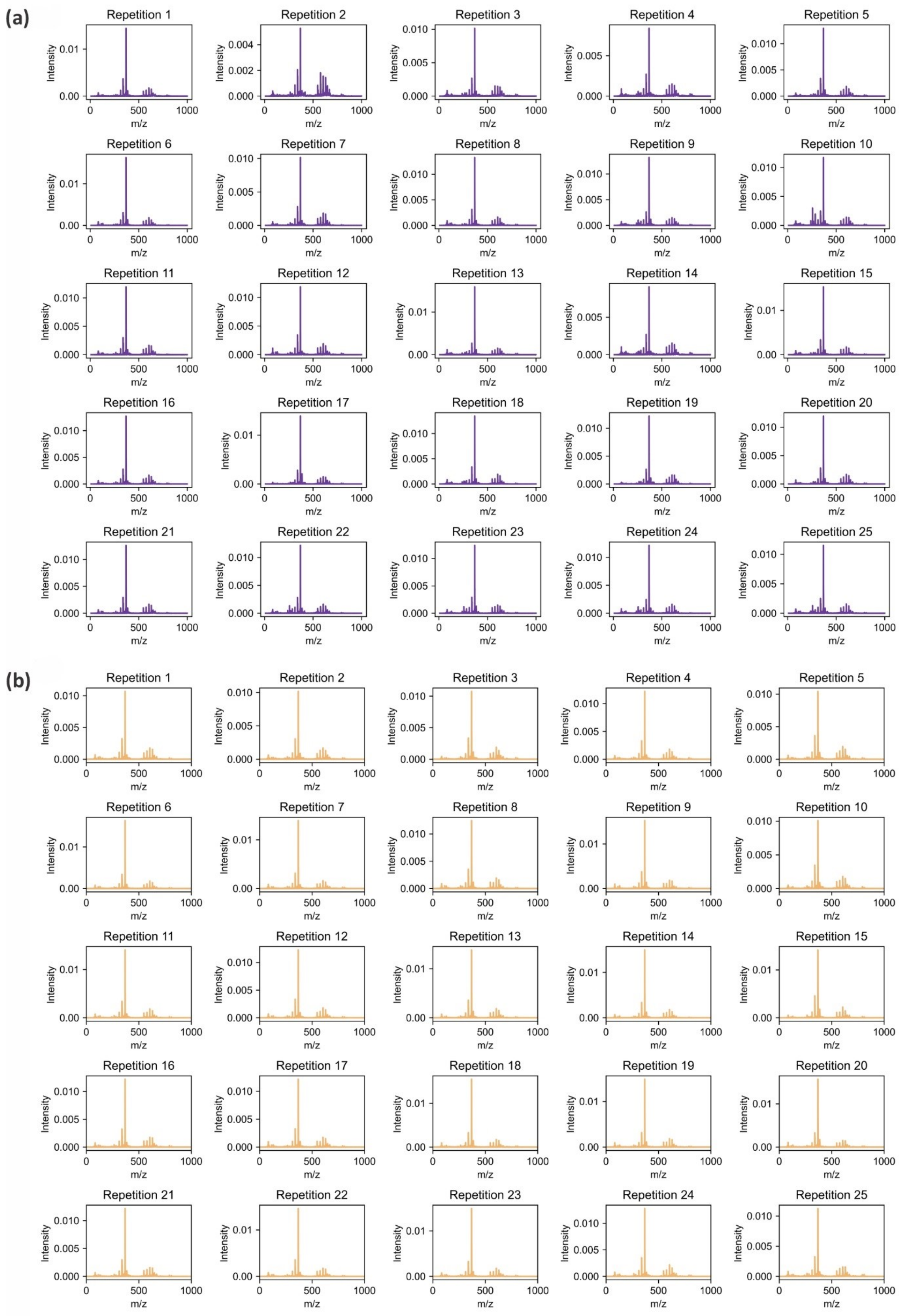

Figure S6: Mass spectra recorded for a frozen brain sample using: (a) cleaning method 1 (without a hot nitrogen cleaning process between each repetition); and (b) cleaning method 2 (with a hot nitrogen cleaning process between each repetition).

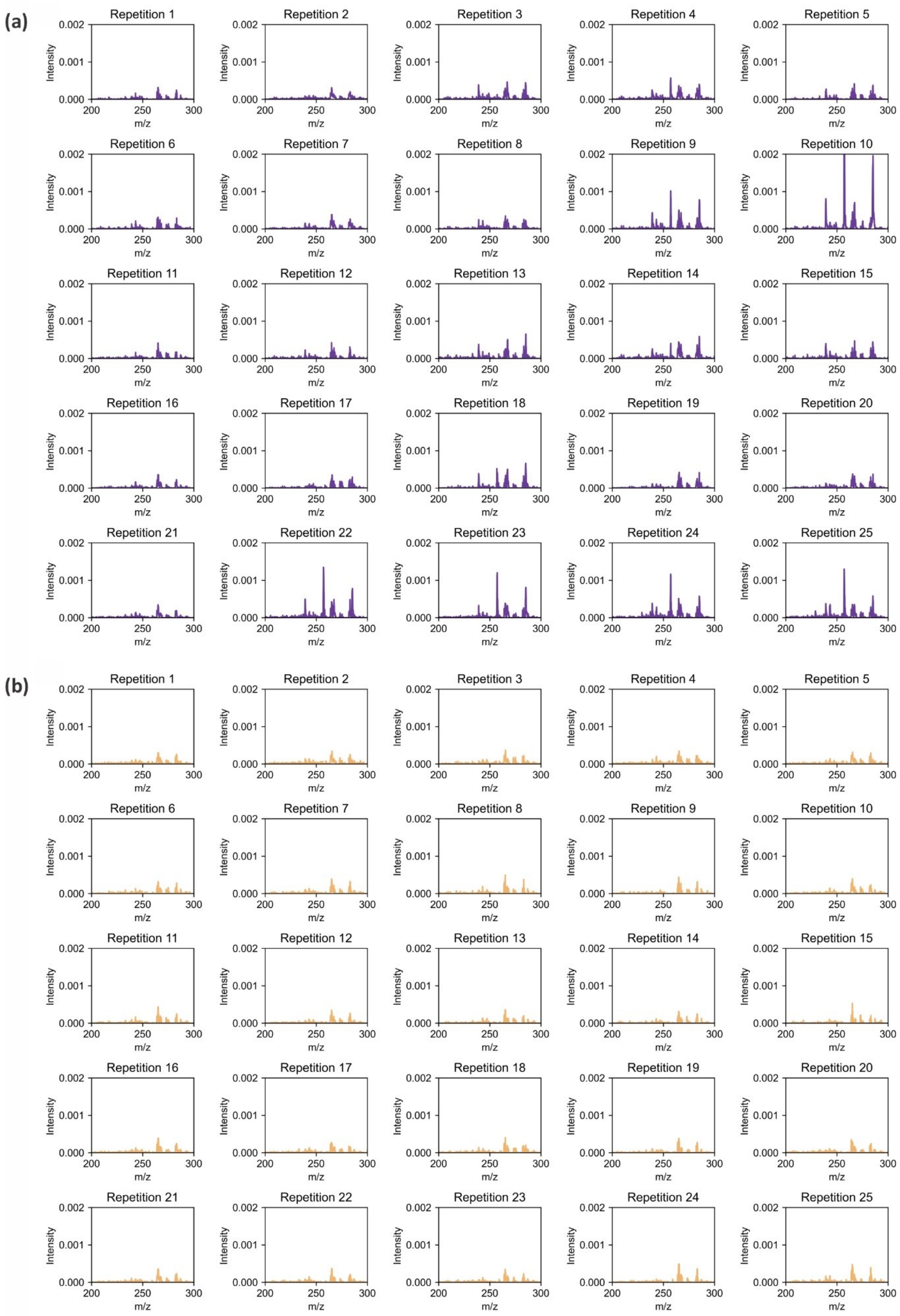

Figure S7: As for Figure S7, but showing only the low mass range between *m*/*z* 200 and 300.

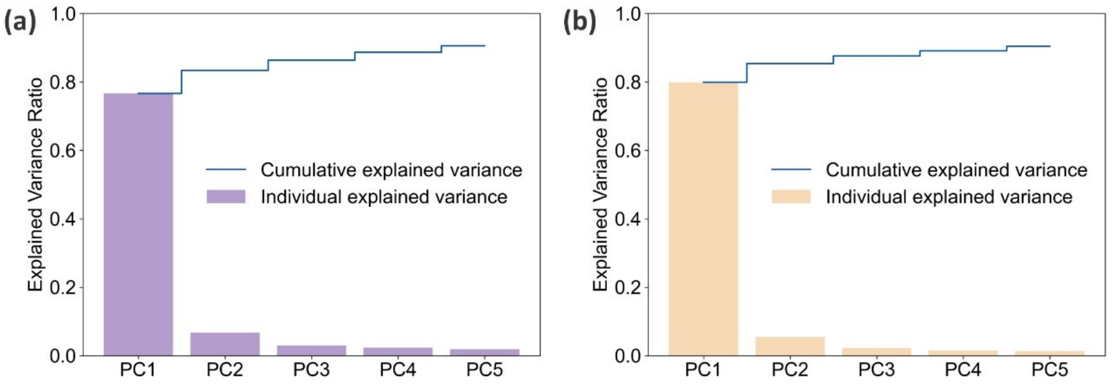

Figure S8: The cumulative explained variance plot from the PCA analysis of 25 repeated measurements on the same brain sample using cleaning methods 1 and 2, using (a) the mass range *m*/*z* 10-1000, and (b) the mass range *m*/*z* 200-300. In both cases, the first five principal components (PCs) together explain more than 90% of the variance.

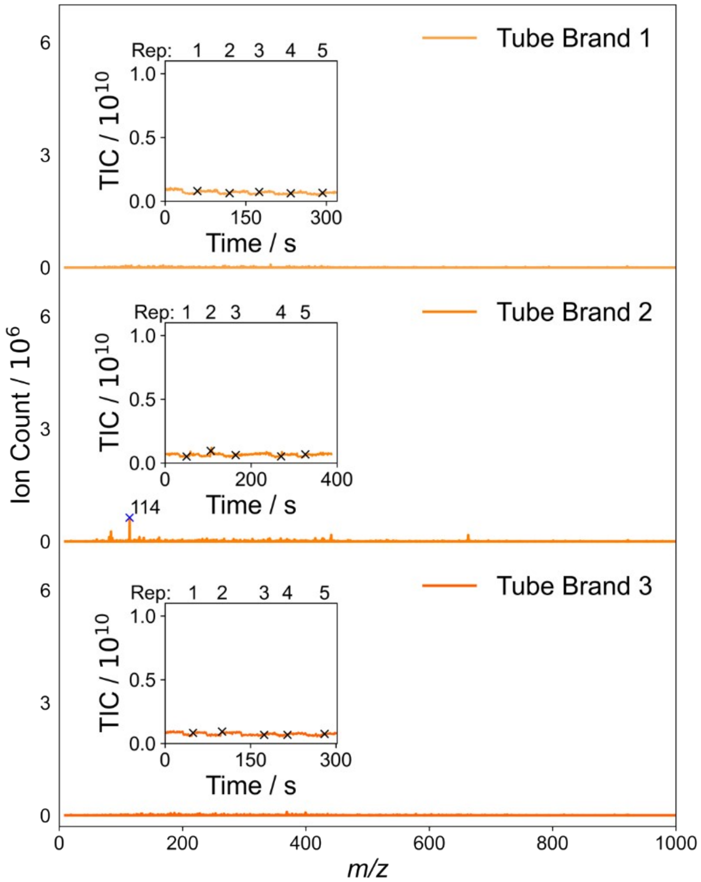

Figure S9: Mass spectra recorded for samples of LC-MS water left overnight in three different brands of sample tube, with insets showing chromatograms for the total ion count (TIC) across five repetitions (Reps). While substantial signal was observed when sampling directly from the walls of the tubes (see main article), there appears to be no signficant leaching of material into solution, with no obvious peaks in the chromatograms and very low signal in the mass spectra.

Table S1: OMNI Homogeniser settings

Parameter	Value
Tube volume / mL	1.5
Speed / ms–1	4.00
Cycles	2
Time / s	30
Dwell / s	15

Table S2: ASAP-MS parameter settings.

Scan settings	
Starting m/z	10
Finishing m/z	1000
Scan time / ms	900
Scan delay / µs	100
**Smoothing**	
Radius	7
Iterations	0
Remove noise	TRUE
**Ion Source settings in 'high temperature low fragmentation, positive ion' mode**	
Polarity	Positive
Capillary Temperature / °C	250
Capillary Voltage / V	120
Source Voltage Offset / V	20
Source Voltage Span / V	0
Source Gas Temperature / °C	400
Transfer Line Temperature / °C	100
APCI Corona Discharge / µA	5

Table S3: FLIR C3-X Compact Thermal Camera settings

Parameter	Value
Emissivity	0.85
Distance	1.00
Atmospheric Temperature / °C	23
Relative humidity	50
Image Scale maximum / °C	85
Image Scale minimum / °C	25

Table S4: Data distribution analysis and statistical significance test method choice

Dataset	Shapiro-Wilk test			Statistical significance test
	Statistic	df	p-value	Method
**Background Effects**				
Contaminated background	0.8573	25	0.002430	Mann-Whitney U test
Clean background	0.9620	25	1.03E-05	
**Glass Capillary Cleaning**				
Method 1 (Whole spectra)	0.9557	25	0.335960	Mann-Whitney U test
Method 2 (Whole spectra)	0.8718	25	0.004693	
Method 1 (m/z 200-300)	0.8689	25	0.004108	Mann-Whitney U test
Method 2 (m/z 200-300)	0.9617	25	0.448487	
**Lens Tissue Peak Test**				
Peak258 (Method 1)	0.6513	25	1.72E-06	Mann-Whitney U test
Peak258 (Method 2)	1.0000	25	1.000000	
Peak275 (Method 1)	0.9260	25	0.070427	Mann-Whitney U test
Peak275 (Method 2)	0.9396	25	0.145646	
Peak285 (Method 1)	0.8301	25	0.000750	Mann-Whitney U test
Peak285 (Method 2)	0.7676	25	6.86E-05	
**User Repeatability**				
User 1 to User 1 Centroid	0.8454	10	0.051221	ANOVA and Tukey's HSD
User 2 to User 2 Centroid	0.8543	10	0.065274	
User 3 to User 3 Centroid	0.9409	10	0.563233	
User 4 to User 4 Centroid	0.8734	10	0.109352	
**User Reproducibility**				
User 1 to User 1 Centroid	0.8454	10	0.051221	ANOVA and Tukey's HSD
User 1 to User 2 Centroid	0.9571	10	0.752081	
User 1 to User 3 Centroid	0.8136	10	0.021196	
User 1 to User 4 Centroid	0.9374	10	0.524356	
User 2 to User 1 Centroid	0.7920	10	0.011601	ANOVA and Tukey's HSD
User 2 to User 2 Centroid	0.8543	10	0.065274	
User 2 to User 3 Centroid	0.9482	10	0.647420	
User 2 to User 4 Centroid	0.9598	10	0.784061	
User 3 to User 1 Centroid	0.9430	10	0.586380	ANOVA and Tukey's HSD
User 3 to User 2 Centroid	0.9739	10	0.924502	
User 3 to User 3 Centroid	0.9409	10	0.563233	
User 3 to User 4 Centroid	0.9756	10	0.937322	
User 4 to User 1 Centroid	0.8941	10	0.188340	ANOVA and Tukey's HSD
User 4 to User 2 Centroid	0.8084	10	0.018355	
User 4 to User 3 Centroid	0.9418	10	0.573641	
User 4 to User 4 Centroid	0.8734	10	0.109352	

* p value < 0.001, data deviation is likely severe; 0.001 < p value ≤ 0.01, data deviation is significant; 0.01 < p value ≤ 0.05, the deviation is moderate; p value > 0.05, data are normally distributed. ANOVA and Tukey's HSD are relatively robust to moderate deviations from normality,[1] so we still applied this method to analyse user reproducibility.

[1] Blanca Mena M J, Alarcón Postigo R, Arnau Gras J, et al. Non-normal data: Is ANOVA still a valid option?[J]. Psicothema, 2017, vol. 29, num. 4, p. 552-557, 2017.

Further analysis of features responsible for batch effects

In order to identify the features responsible for the separation caused by batch effects and observed along the first principal component (PC1) in Figure 6, we examined the PC1 loadings, which represent the contribution of each original variable to the principal component. Features (*m/z* peaks) with higher absolute loading values were interpreted as having a greater impact on the direction of PC1. The eight most significant mass peaks according to their PC1 loadings are shown in Figure S10, with their intensity distributions within each batch shown in the form of box and whisker plots in Figure S11.

There is a strong correlation between the absolute intensity of the *m/z* peaks and their contribution to batch effects, with the most intense features in the mass spectra playing the largest role. Unsurprisingly, any small technical differences between batches can cause noticeable changes in the most intense peaks. Intensity changes in less intense peaks are then induced either directly via the technical differences or as a secondary effect of the intense peak variation, via the normalisation procedure employed as part of the data pre-processing. The more intense peaks therefore make the most significant contributions to the observed batch effects.

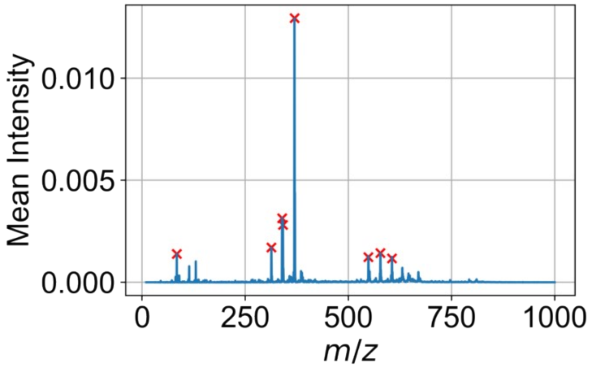

Figure S10: The eight peaks with the highest loadings for PC1 in the analysis of batch effects, marked with red crosses. The spectrum shown in blue is the average across all samples.

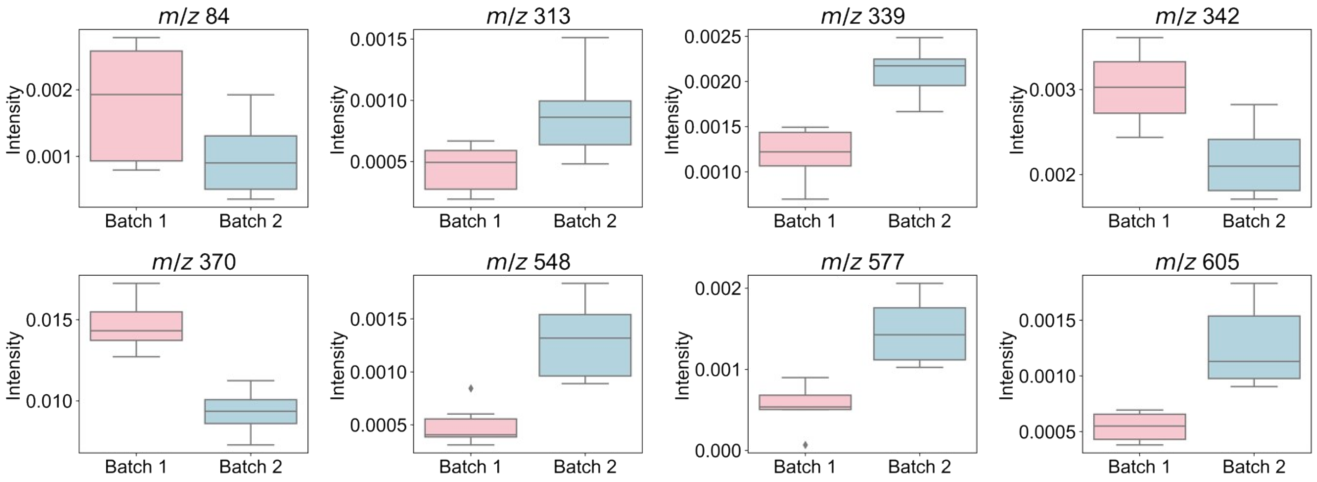

Figure S11: Box and whisker plots of the intensity distributions within each batch for each of the peaks identified in Figure S10.
